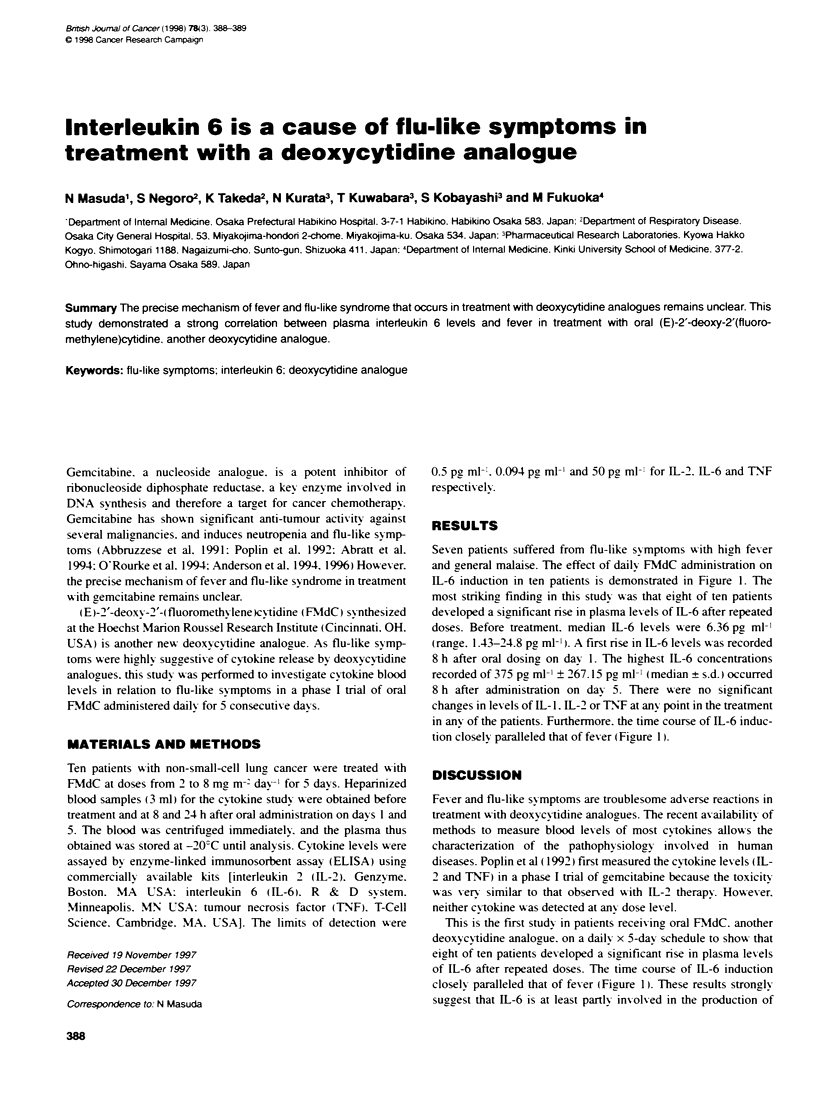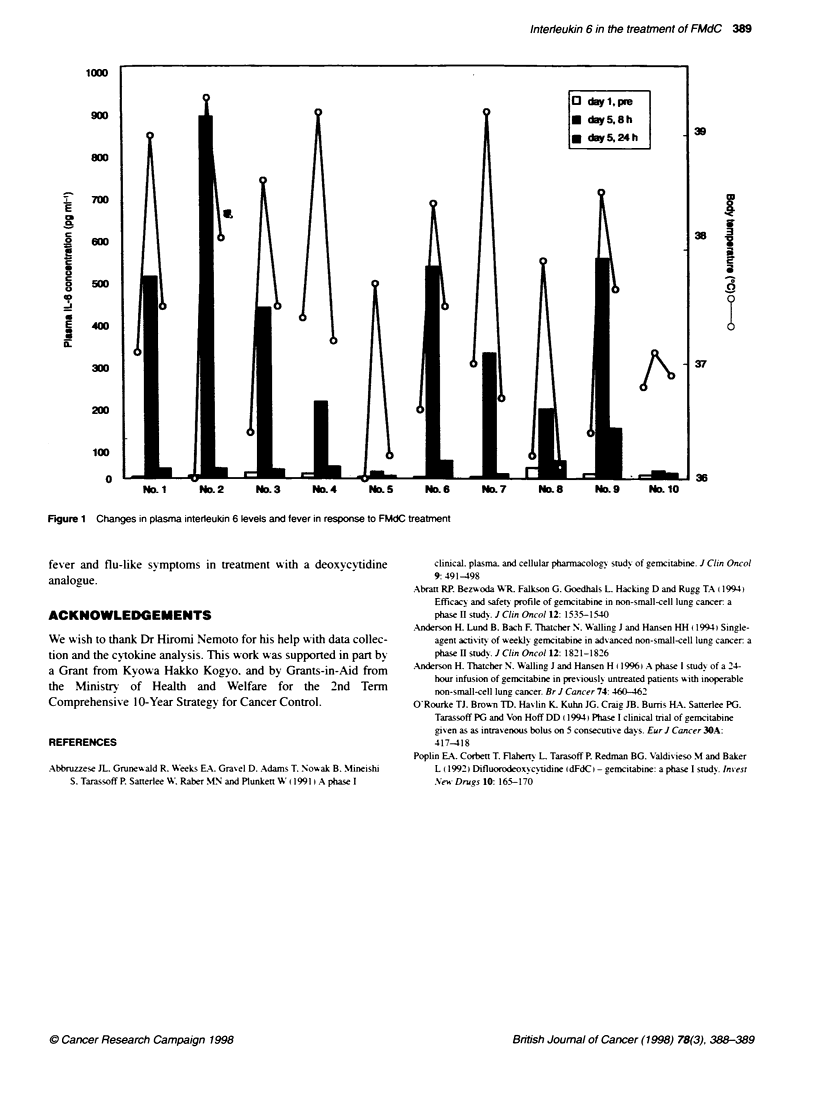# Interleukin 6 is a cause of flu-like symptoms in treatment with a deoxycytidine analogue.

**DOI:** 10.1038/bjc.1998.504

**Published:** 1998-08

**Authors:** N. Masuda, S. Negoro, K. Takeda, N. Kurata, T. Kuwabara, S. Kobayashi, M. Fukuoka

**Affiliations:** Department of Internal Medicine, Osaka Prefectural Habikino Hospital, Japan.

## Abstract

The precise mechanism of fever and flu-like syndrome that occurs in treatment with deoxycytidine analogues remains unclear. This study demonstrated a strong correlation between plasma interleukin 6 levels and fever in treatment with oral (E)-2'-deoxy-2'(fluoromethylene)cytidine, another deoxycytidine analogue.


British Journal of Cancer (1998) 78(3), 388–389
© 1998 Cancer Research Campaign

### Interleukin 6 is a cause of flu-like symptoms in treatment with a deoxycytidine analogue

**N Masuda[1], S Negoro[2], K Takeda[2], N Kurata[3], T Kuwabara[3], S Kobayashi[3] and M Fukuoka[4]**

[1]Department of Internal Medicine, Osaka Prefectural Habikino Hospital, 3-7-1 Habikino, Habikino Osaka 583, Japan; [2]Department of Respiratory Disease, Osaka City General Hospital, 53, Miyakojima-hondori 2-chome, Miyakojima-ku, Osaka 534, Japan; [3]Pharmaceutical Research Laboratories, Kyowa Hakko Kogyo, Shimotogari 1188, Nagaizumi-cho, Sunto-gun, Shizuoka 411, Japan; [4]Department of Internal Medicine, Kinki University School of Medicine, 377-2, Ohno-higashi, Sayama Osaka 589, Japan

**Summary** The precise mechanism of fever and flu-like syndrome that occurs in treatment with deoxycytidine analogues remains unclear. This study demonstrated a strong correlation between plasma interleukin 6 levels and fever in treatment with oral (E)-2′-deoxy-2′(fluoromethylene)cytidine, another deoxycytidine analogue.

**Keywords:** flu-like symptoms; interleukin 6; deoxycytidine analogue

Gemcitabine, a nucleoside analogue, is a potent inhibitor of ribonucleoside diphosphate reductase, a key enzyme involved in DNA synthesis and therefore a target for cancer chemotherapy. Gemcitabine has shown significant anti-tumour activity against several malignancies, and induces neutropenia and flu-like symptoms (Abbruzzese et al, 1991; Poplin et al, 1992; Abratt et al, 1994; O'Rourke et al, 1994; Anderson et al, 1994, 1996) However, the precise mechanism of fever and flu-like syndrome in treatment with gemcitabine remains unclear.

(E)-2′-deoxy-2′-(fluoromethylene)cytidine (FMdC) synthesized at the Hoechst Marion Roussel Research Institute (Cincinnati, OH, USA) is another new deoxycytidine analogue. As flu-like symptoms were highly suggestive of cytokine release by deoxycytidine analogues, this study was performed to investigate cytokine blood levels in relation to flu-like symptoms in a phase I trial of oral FMdC administered daily for 5 consecutive days.

### MATERIALS AND METHODS

Ten patients with non-small-cell lung cancer were treated with FMdC at doses from 2 to 8 mg $m^{-2}$ $day^{-1}$ for 5 days. Heparinized blood samples (3 ml) for the cytokine study were obtained before treatment and at 8 and 24 h after oral administration on days 1 and 5. The blood was centrifuged immediately, and the plasma thus obtained was stored at –20°C until analysis. Cytokine levels were assayed by enzyme-linked immunosorbent assay (ELISA) using commercially available kits [interleukin 2 (IL-2), Genzyme, Boston, MA USA; interleukin 6 (IL-6), R & D system, Minneapolis, MN USA; tumour necrosis factor (TNF), T-Cell Science, Cambridge, MA, USA]. The limits of detection were 0.5 pg $ml^{-1}$, 0.094 pg $ml^{-1}$ and 50 pg $ml^{-1}$ for IL-2, IL-6 and TNF respectively.

*Received 19 November 1997*
*Revised 22 December 1997*
*Accepted 30 December 1997*

*Correspondence to:* N Masuda

### RESULTS

Seven patients suffered from flu-like symptoms with high fever and general malaise. The effect of daily FMdC administration on IL-6 induction in ten patients is demonstrated in Figure 1. The most striking finding in this study was that eight of ten patients developed a significant rise in plasma levels of IL-6 after repeated doses. Before treatment, median IL-6 levels were 6.36 pg $ml^{-1}$ (range, 1.43–24.8 pg $ml^{-1}$). A first rise in IL-6 levels was recorded 8 h after oral dosing on day 1. The highest IL-6 concentrations recorded of 375 pg $ml^{-1}$ ± 267.15 pg $ml^{-1}$ (median ± s.d.) occurred 8 h after administration on day 5. There were no significant changes in levels of IL-1, IL-2 or TNF at any point in the treatment in any of the patients. Furthermore, the time course of IL-6 induction closely paralleled that of fever (Figure 1).

### DISCUSSION

Fever and flu-like symptoms are troublesome adverse reactions in treatment with deoxycytidine analogues. The recent availability of methods to measure blood levels of most cytokines allows the characterization of the pathophysiology involved in human diseases. Poplin et al (1992) first measured the cytokine levels (IL-2 and TNF) in a phase I trial of gemcitabine because the toxicity was very similar to that observed with IL-2 therapy. However, neither cytokine was detected at any dose level.

This is the first study in patients receiving oral FMdC, another deoxycytidine analogue, on a daily × 5-day schedule to show that eight of ten patients developed a significant rise in plasma levels of IL-6 after repeated doses. The time course of IL-6 induction closely paralleled that of fever (Figure 1). These results strongly suggest that IL-6 is at least partly involved in the production of

**Figure 1** Changes in plasma interleukin 6 levels and fever in response to FMdC treatment fever and flu-like symptoms in treatment with a deoxycytidine analogue.

### ACKNOWLEDGEMENTS

We wish to thank Dr Hiromi Nemoto for his help with data collection and the cytokine analysis. This work was supported in part by a Grant from Kyowa Hakko Kogyo, and by Grants-in-Aid from the Ministry of Health and Welfare for the 2nd Term Comprehensive 10-Year Strategy for Cancer Control.

### REFERENCES

Abbruzzese JL, Grunewald R, Weeks EA, Gravel D, Adams T, Nowak B, Mineishi S, Tarassoff P, Satterlee W, Raber MN and Plunkett W (1991) A phase I clinical, plasma, and cellular pharmacology study of gemcitabine. *J Clin Oncol* **9**: 491–498

Abratt RP, Bezwoda WR, Falkson G, Goedhals L, Hacking D and Rugg TA (1994) Efficacy and safety profile of gemcitabine in non-small-cell lung cancer: a phase II study. *J Clin Oncol* **12**: 1535–1540

Anderson H, Lund B, Bach F, Thatcher N, Walling J and Hansen HH (1994) Single-agent activity of weekly gemcitabine in advanced non-small-cell lung cancer: a phase II study. *J Clin Oncol* **12**: 1821–1826

Anderson H, Thatcher N, Walling J and Hansen H (1996) A phase I study of a 24-hour infusion of gemcitabine in previously untreated patients with inoperable non-small-cell lung cancer. *Br J Cancer* **74**: 460–462

O'Rourke TJ, Brown TD, Havlin K, Kuhn JG, Craig JB, Burris HA, Satterlee PG, Tarassoff PG and Von Hoff DD (1994) Phase I clinical trial of gemcitabine given as as intravenous bolus on 5 consecutive days. *Eur J Cancer* **30A**: 417–418

Poplin EA, Corbett T, Flaherty L, Tarasoff P, Redman BG, Valdivieso M and Baker L (1992) Difluorodeoxycytidine (dFdC) – gemcitabine: a phase I study. *Invest New Drugs* **10**: 165–170

© Cancer Research Campaign 1998